# The MASLD–Heart Axis: Biological Mechanisms, Cardiac Remodeling, and Clinical Implications

**DOI:** 10.3390/ph19101629

**Published:** 2026-10-14

**Authors:** Evangelos Oikonomou, Nikolaos P. Machairas, Panagiotis Theofilis, Anna Maria Louka, Maria Xenou, Panagiota K. Stampouloglou, Ourania Katsarou, Nikolaos K. Kokkinos, Antonios Lysandrou, Stergios Karamesinis-Spinthakis, Dimitris Tousoulis, Gerasimos Siasos

**Affiliations:** 1 3rd Cardiology Department, “Sotiria” General Hospital of Chest Diseases of Athens, Athina Ioanni Martinou Endowed Chair; Medical School, National and Kapodistrian University of Athens, 11527 Athens, Greece; 2 First Department of Cardiology, School of Medicine, National and Kapodistrian University of Athens, Hippokration General Hospital, 11527 Athens, Greece; nmaxairas@hotmail.com (N.P.M.); panos.theofilis@hotmail.com (P.T.); drtousoulis@hotmail.com (D.T.); 3 Third Department of Cardiology, “Sotiria” Chest Diseases General Hospital, School of Medicine, National and Kapodistrian University of Athens, 11527 Athens, Greece; dr.louka.annamaria@gmail.com (A.M.L.); xenomary@gmail.com (M.X.); stampoulogloupanagiota@gmail.com (P.K.S.); raniakatsarou@yahoo.gr (O.K.); nikoskokkinoss3@hotmail.gr (N.K.K.); ant.lysandrou@hotmail.com (A.L.); sterkaraspi@gmail.com (S.K.-S.); gsiasos@med.uoa.gr (G.S.)

**Keywords:** metabolic dysfunction-associated steatotic liver disease, cardiovascular disease, atherosclerosis, heart failure, endothelial dysfunction, ventricular remodeling

## Abstract

**Background/Objectives**: Metabolic dysfunction-associated steatotic liver disease (MASLD), the hepatic manifestation of cardiometabolic dysfunction, is closely linked to atherosclerosis, heart failure, and adverse cardiovascular outcomes. This review summarizes current evidence on the biological basis of the MASLD–heart axis, with particular emphasis on vascular injury and adverse cardiac remodeling. **Methods**: A narrative review of contemporary literature was conducted, including experimental studies, observational cohorts, meta-analyses, randomized clinical trials, and current guideline recommendations addressing the cardiohepatic axis. **Results**: The interaction between the liver and the heart involves interconnected mechanisms, including insulin resistance, lipotoxicity, oxidative stress, chronic inflammation, endothelial dysfunction, and neurohormonal activation. Experimental and translational evidence supports biologically plausible links between MASLD-related metabolic and inflammatory pathways and vascular or myocardial injury, while clinical studies consistently associate MASLD with arterial stiffness, microvascular dysfunction, atherosclerotic disease, adverse cardiac remodeling, heart failure, atrial fibrillation, and cardiovascular events. Several of these associations persist after adjustment for conventional cardiometabolic risk factors, although residual confounding and shared risk determinants limit causal inference. Non-invasive tools, including the Fibrosis-4 (FIB-4) score and transient elastography, may help identify patients at higher risk of advanced fibrosis and cardiometabolic complications. Lifestyle interventions remain the cornerstone of management, while glucagon-like peptide-1 receptor agonists, sodium-glucose cotransporter-2 inhibitors, and fibroblast growth factor-21 analogs may provide combined hepatic and cardiovascular benefits. **Conclusions**: The cardiohepatic axis is an important component of cardiometabolic disease, linking hepatic dysfunction with vascular and myocardial injury through shared biological pathways. Incorporating hepatic assessment into cardiovascular risk evaluation may support earlier identification of high-risk patients.

## 1. Introduction

Cardiovascular diseases (CVD) remain the leading cause of morbidity and mortality worldwide, driven by complex and multifactorial pathophysiology [1]. Among the major contributors to CVD progression, the liver has recently gained attention as a key component through its complex interplay with the heart. The liver–heart axis reflects the convergence of hemodynamic pathways, vascular dysfunction, metabolic dysregulation, and chronic low-grade inflammation in a vicious cycle where both organs contribute to disease progression; many of the underlying mechanisms remain under investigation.

The liver acts as a central metabolic core, regulating lipid synthesis and transport, glucose homeostasis, coagulation pathways, and inflammatory signaling, thereby exerting systemic effects that directly influence vascular integrity and cardiac remodeling [2]. Dysregulation of these pathways promotes atherogenesis, endothelial dysfunction, myocardial fibrosis, and, eventually, altered myocardial metabolism [3]. Disruptions in the liver–heart interactions contribute to the pathogenesis of CVD, especially in the settings of cardiometabolic disorders.

Steatotic liver disease (SLD) is the overarching term encompassing the different etiologies of hepatic steatosis. According to the 2023 multi-society consensus, metabolic dysfunction-associated steatotic liver disease (MASLD) is defined by hepatic steatosis together with at least one of five cardiometabolic risk factors: (1) overweight/obesity, defined as body mass index ≥ 25 kg/m^2^ (≥23 kg/m^2^ in Asian individuals) or increased waist circumference (>94 cm in men and >80 cm in women, with ethnicity-adjusted thresholds where appropriate); (2) dysglycemia, defined as fasting glucose ≥ 100 mg/dL, 2 h post-load glucose ≥ 140 mg/dL, HbA1c ≥ 5.7%, established type 2 diabetes, or treatment for type 2 diabetes; (3) blood pressure ≥ 130/85 mmHg or antihypertensive treatment; (4) plasma triglycerides ≥ 150 mg/dL or lipid-lowering treatment; or (5) low HDL cholesterol, defined as ≤40 mg/dL in men or ≤50 mg/dL in women, or lipid-lowering treatment (PMID 38851997). Subjects with MASLD have a higher risk of cardiovascular events, although the magnitude of this association varies according to the population studied, MASLD severity and fibrosis stage, comparator group, and cardiovascular outcome assessed [4,5]. In several observational analyses, the association between MASLD and cardiovascular risk persists after adjustment for selected conventional cardiometabolic risk factors; however, shared determinants and residual confounding limit causal interpretation [6]. MALD is also closely associated with atherogenic dyslipidemia and hypertension, both of which amplify CVD risk [7]. Importantly, MASLD is an affirmative diagnosis based on metabolic dysfunction and may coexist with other etiologies of liver disease.

Within this framework, metabolic dysfunction-associated steatohepatitis (MASH) denotes MASLD with steatohepatitis. Individuals fulfilling MASLD criteria who consume 140–350 g of alcohol per week for women or 210–420 g per week for men are classified as having metabolic dysfunction- and alcohol-associated liver disease (MetALD), reflecting the overlapping contributions of metabolic dysfunction and alcohol exposure. In contrast, individuals with hepatic steatosis who have neither cardiometabolic risk factors nor another identifiable cause are classified as having cryptogenic SLD. Although MASLD and MASH represent the preferred contemporary terminology, much of the available evidence predates the 2023 nomenclature change and was generated using the terms non-alcoholic fatty liver disease (NAFLD) and non-alcoholic steatohepatitis (NASH). Contemporary analyses have demonstrated very high concordance between the historical NAFLD and current MASLD definitions, supporting the general applicability of legacy NAFLD evidence to MASLD populations [8,9]. Accordingly, MASLD/MASH are preferentially used in the general narrative, whereas NAFLD, NASH, or fatty liver disease (FLD) are retained when describing individual studies according to the terminology and diagnostic criteria originally used.

This review examines recent evidence on the MASLD–heart axis and its clinical implications for CVD, focusing on shared biological mechanisms, vascular and cardiac remodeling, risk assessment, diagnostic strategies, and emerging therapeutic approaches. It further highlights the bidirectional interactions between metabolic liver disease and cardiovascular dysfunction while maintaining a specific focus on MASLD rather than the broader spectrum of liver–heart interaction.

## 2. Literature Search

PubMed/MEDLINE, Scopus, Web of Science, and Google Scholar were searched from database inception through June 15, 2026. Search terms included combinations of “metabolic dysfunction-associated steatotic liver disease”, “MASLD”, “metabolic dysfunction-associated steatohepatitis”, “MASH”, “non-alcoholic fatty liver disease”, “NAFLD”, “liver-heart axis”, “cardiovascular disease”, “atherosclerosis”, “endothelial dysfunction”, “inflammation”, “oxidative stress”, “arterial stiffness”, “microvascular dysfunction”, “heart failure”, “cardiac remodeling”, “steatosis”, and “fibrosis.” Reference lists of articles were also screened to identify additional relevant publications. No language restrictions were applied.

Original clinical studies, systematic reviews, meta-analyses, randomized clinical trials, and major international clinical practice guidelines or consensus statements were considered. Priority was given to contemporary large-scale observational cohorts, prospective studies, randomized trials, systematic reviews and meta-analyses, and recommendations from major cardiovascular and hepatology societies. Preclinical and translational studies were selectively included when they provided mechanistic insight into metabolic, inflammatory, vascular, or myocardial pathways relevant to the MASLD-heart axis. Earlier seminal studies were retained when considered necessary to describe established biological mechanisms.

Study selection was performed according to relevance to the predefined thematic areas of the review including metabolic and inflammatory mechanisms, vascular dysfunction, cardiac remodeling, cardiovascular risk assessment, and therapeutic implications, rather than through a formal systematic review framework. Accordingly, no formal risk of bias assessment or quantitative evidence synthesis was undertaken. Studies were selected when they specifically evaluated associations between MASLD, hepatic steatosis, or liver fibrosis and objectively assessed cardiac structural or functional abnormalities. When multiple studies addressed overlapping questions, preference was given to larger and better-characterized cohorts, longitudinal investigations, studies using validated imaging or histological methods for liver assessment, and contemporary systematic reviews or meta-analyses.

## 3. Biological Basis of the Liver–Heart Axis

### 3.1. Shared Metabolic Pathways

**Figure 1:**
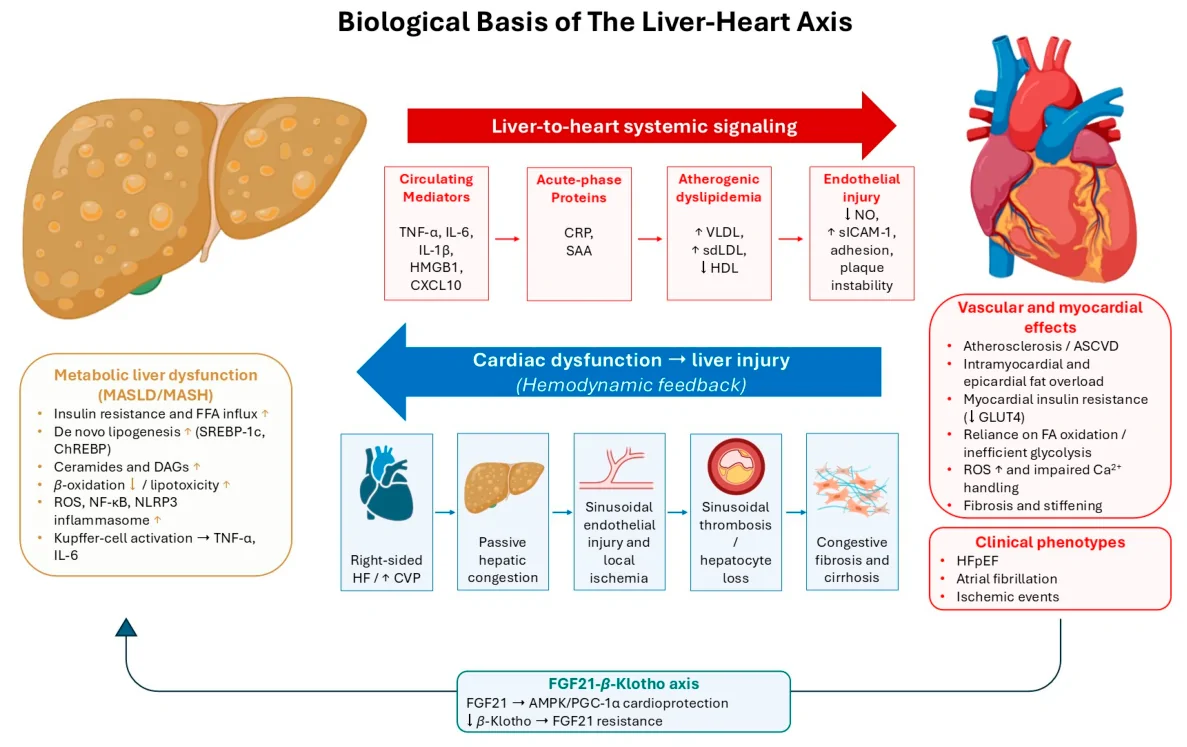
Biological mechanisms underlying the liver–heart axis. Metabolic liver dysfunction promotes systemic inflammation, atherogenic dyslipidemia, endothelial dysfunction, and myocardial metabolic remodeling, resulting in atherosclerosis, HFpEF, atrial fibrillation, and ischemic cardiovascular disease. Conversely, cardiac dysfunction exacerbates liver injury through venous congestion, sinusoidal ischemia, thrombosis, and fibrosis, establishing a bidirectional liver–heart feedback loop. The FGF21/β-Klotho axis may partially counteract these processes by preserving myocardial energy homeostasis.

The Liver–Heart Axis plays a pivotal role in coordinating systemic metabolic and hemodynamic homeostasis. The heart is metabolically flexible and uses multiple circulating substrates, including fatty acids, glucose, lactate, ketone bodies, and amino acids, to meet its high ATP demands. The liver contributes importantly to this metabolic environment by regulating glucose and lipid metabolism and by producing ketone bodies during fasting and other states of increased metabolic demand [10,11]. The complex bidirectional communication between the liver and the heart is mediated through intertwined metabolic, inflammatory, vascular, and hemodynamic pathways, which are summarized in Figure 1.

The Sympathetic Nervous System and Renin–Angiotensin–Aldosterone System (RAAS) modulate metabolism during stress, ensuring the synchronization of hepatic gluconeogenesis and lipolysis with cardiac output requirements [12,13,14]. In addition, the liver plays a central role in systemic lipid homeostasis. Preclinical studies support an important role for Peroxisome Proliferator-Activated Receptor alpha (PPAR-α) in hepatic fatty-acid metabolism, with PPAR-α deficiency aggravating metabolic and atherosclerotic abnormalities in experimental models [15]. Hepatic lipid homeostasis reflects a balance among de novo lipogenesis very-low-density lipoprotein (VLDL) secretion, receptor-mediated clearance of lipoprotein remnants via Low Density Lipoprotein Receptor (LDLR) and Apolipoprotein E (ApoE), and pathways involved in reverse cholesterol transport through High Density Lipoprotein (HDL) [16,17,18,19,20]. In clinical populations, MASLD is frequently accompanied by an atherogenic lipid profile characterized by hypertriglyceridemia, increased triglyceride-rich lipoproteins and small dense LDL particles, and reduced HDL cholesterol, providing a potential metabolic link between hepatic lipid dysregulation and cardiovascular risk [21].

Moreover, metabolic inflexibility, accompanying insulin resistance, obesity, MASLD, and heart failure (HF), particularly HFpEF, is characterized by an impaired ability to switch between glucose, fatty acids, and ketone bodies according to metabolic demands [22]. This metabolic phenotype is closely linked to insulin resistance and mitochondrial dysfunction, leading to excessive hepatic glucose production, increased free fatty acid flux, and enhanced VLDL secretion. The resulting oversupply of circulating metabolic substrates promotes myocardial substrate overload, impaired mitochondrial oxidative metabolism, and accumulation of toxic lipid intermediates. Consequently, the failing heart exhibits reduced metabolic flexibility, diminished ATP production, and progressive lipotoxic injury [11]. Similar disturbances in substrate utilization are also observed in HFrEF, although the pattern of metabolic adaptation varies according to HF phenotype and disease stage. In HFrEF, particularly in advanced disease, human and experimental studies support increased myocardial ketone uptake and oxidation as an adaptive shift in substrate utilization. In contrast, ketone metabolism in HFpEF is less well characterized, and increased circulating ketone concentrations do not necessarily imply increased myocardial ketone oxidation [23,24,25].

### 3.2. Insulin Resistance, Lipotoxicity, Oxidative Stress

The interaction between liver and heart may be described by a “multiple parallel-hit” model comprising insulin resistance (IR), lipotoxicity, and oxidative stress [26,27]. Hepatic insulin resistance is characterized by persistent hepatic gluconeogenesis despite hyperinsulinemia, whereas insulin-stimulated de novo lipogenesis (DNL) is preserved or enhanced through activation of Sterol Regulatory Element-Binding Protein 1c (SREBP-1c) and Carbohydrate-Responsive Element-Binding Protein (ChREBP) [28]. This condition is exacerbated by impaired suppression of adipose lipolysis and reduced skeletal muscle glucose uptake, resulting in increased hepatic delivery of FFAs [29,30,31]. Under insulin-resistant conditions, the normal insulin-mediated suppression of ApoB-containing VLDL secretion becomes impaired, while increased FFA delivery and enhanced de novo lipogenesis provides additional triglyceride substrate for VLDL assembly. These changes favor increased hepatic VLDL secretion and contribute to the atherogenic dyslipidemia characteristic of insulin resistance [32].

Chronic nutrient overload may promote lipotoxicity, a state associated with overwhelmed fatty acid processing and the inactivation of Forkhead Box A2 (FOXA2), which collectively may impair mitochondrial β-oxidation [33]. Hepatic accumulation of diacylglycerols (DAGs) has been linked primarily to activation of Protein Kinase C epsilon (PKC-ε), which can impair insulin-receptor signaling and represents one of the best-characterized mechanisms of lipid-induced hepatic insulin resistance. In contrast, PKC-θ is more strongly implicated in DAG-mediated insulin resistance in skeletal muscle, whereas the contribution of PKC-δ to hepatic insulin resistance appears more context-dependent [34,35]. These perturbations activate inflammatory cascades involving inhibitor of nuclear factor kappa-B kinase subunit beta (IKK-β), c-Jun N-terminal kinase (JNK), and M1-polarized kupffer cells, which release cytokines, such as tumor necrosis factor alpha (TNF-α) and interleukin 6 (IL-6), that may contribute to hepatic fibrogenesis and systemic endothelial dysfunction. Preclinical and translational studies support the involvement of these pathways in hepatic fibrogenesis and systemic endothelial dysfunction, providing biological plausibility for downstream cardiovascular injury [36].

Notably, metabolic dysfunction may be accompanied by abnormalities in distinct cardiac lipid compartments. Epicardial adipose tissue represents a visceral fat depot located between the myocardium and visceral pericardium and may influence adjacent myocardium and coronary vessels through local paracrine and inflammatory signaling. In contrast, intramyocardial triglyceride accumulation, or myocardial steatosis, reflects intracellular lipid storage within cardiomyocytes and may be associated with altered myocardial metabolism and function [37,38]. Concurrently, myocardial IR, marked by reduced sarcolemmal glucose transporter type 4 (GLUT4) availability, may favor greater reliance on fatty acid oxidation, as suggested by preclinical models [11,39,40]. This metabolic shift increases reactive oxygen species (ROS) production and impairs calcium signaling, providing a potential mechanistic link to impaired cardiomyocyte relaxation and diastolic dysfunction [41].

Oxidative stress, characterized by a pathological imbalance between the production of ROS and antioxidant defense mechanisms, is a key factor in MASLD and its systemic consequences [42]. While basal ROS levels are vital for normal redox signaling, regulating metabolism, immune responses, and epigenetics, excessive ROS production, primarily through mitochondrial fatty acid over-oxidation and the cytochrome P450 2E1 (CYP2E1) pathway, leads to cellular damage [43]. In the liver, this oxidative environment causes lipid peroxidation and activates the nuclear factor kappa-light-chain-enhancer of activated B cells (NF-κB) pathway, promoting the progression from simple steatosis to metabolic dysfunction-associated steatohepatitis (MASH) [44,45].

Beyond hepatic injury, the metabolically stressed liver may contribute to systemic inflammation through the release of mediators such as TNF-α, IL-6, high-mobility group box 1 (HMGB1), chemokines including C-X-C motif chemokine ligand 10 (CXCL10), and adhesion molecules such as soluble intercellular adhesion molecule-1 (sICAM-1). HMGB1, although primarily an intracellular protein, can act as a pro-inflammatory danger signal when released extracellularly [46]. This “multiple-hit” inflammatory milieu, coupled with the systemic effects of insulin resistance and hyperglycemia-induced advanced glycation end-products (AGEs), may contribute to coronary endothelial dysfunction, adverse extracellular-matrix remodeling, and to plaque instability [47,48]. These integrated pathways potentially modulated by factors such as gut-derived ethanol and circadian disruption may impair myocardial energy homeostasis and ventricular compliance and thereby provide mechanistic links to atherosclerotic cardiovascular disease (ASCVD) and HFpEF; however, their relative contribution to these complex clinical phenotypes in humans remains uncertain [49,50,51].

### 3.3. Inflammation and Cytokine Signaling

The liver is an immunologically active organ in which resident immune cells and hepatic parenchymal and non-parenchymal cells coordinate homeostatic and inflammatory responses [52,53,54]. Resident Kupffer cells, in response to pathogen-associated or damage-associated molecular patterns (PAMPs/DAMPs), can become activated and release pro-inflammatory cytokines such as TNF-α, IL-1, and IL-6 [53,55,56]. Concurrently, metabolic stress may induce hepatocytes to release extracellular vesicles and danger signals, while activation of hepatic stellate cells (HSCs) contributes to chemokine signaling and extracellular-matrix remodeling [57,58]. Hepatic production of acute-phase proteins (APPs), including C-reactive protein (CRP) and serum amyloid A (SAA), is regulated in part by IL-6-family signaling through the gp130–STAT3 pathway [59,60]. Furthermore, preclinical and translational studies implicate activation of the NLRP3 inflammasome in caspase-1-dependent maturation of IL-1β and IL-18 and in pyroptotic signaling during liver injury [61,62]. This coordinated cascade upregulates endothelial adhesion molecules, prompting the rapid recruitment of circulating neutrophils and monocytes to the hepatic microenvironment [53,63]. Sustained hepatic inflammation, although initially acting as a protective response to neutralize threats, can potentially lead to chronic oxidative stress and tissue remodeling, transitioning benign wound healing into progressive fibrosis and cirrhosis.

Experimental and translational evidence supports several biologically plausible pathways through which the metabolic and inflammatory milieu associated with MASLD may contribute to atherogenesis, including lipotoxicity, chronic hepatic injury, and systemic inflammatory signaling [64]. Peripheral insulin resistance increases hepatic exposure to excess free fatty acids, favoring lipotoxicity and oxidative stress that may promote hepatocyte injury. This localized stress activates resident kupffer cells and recruits circulating macrophages to release pro-inflammatory cytokines (IL-1β, IL-6, TNF-α) and CRP, potentially contributing to hepatic fibrogenesis [65,66,67,68]. Concurrently, hepatic lipase and cholesteryl ester transfer protein (CETP) activities alter lipid metabolism in hepatic dysfunction, generating highly atherogenic small dense LDL (sdLDL) alongside sustained elevation of circulating VLDL [69,70]. Circulating pro-inflammatory mediators and atherogenic lipoproteins may adversely affect endothelial function through reduced nitric oxide bioavailability and increased expression of vascular adhesion molecules [66]. This altered vascular environment facilitate LDL, and its oxidized derivatives, and recruited monocytes to penetrate the arterial intima, where macrophages engulf oxidized LDL to form lipid-laden foam cells, the bedrock of atherosclerotic plaques [66,71]. Persistent inflammatory signaling has also been linked to alterations in matrix metalloproteinases (MMPs) and their tissue inhibitors (TIMPs), pathways that may influence extracellular-matrix remodeling and plaque stability [72,73,74]. Together, these mechanisms provide biological plausibility for the observed association between MASLD and atherosclerotic cardiovascular disease; however, they do not by themselves establish that MASLD directly causes plaque rupture or ischemic events in humans. Cardiovascular disease nevertheless remains a major cause of morbidity and mortality in individuals with MASLD [74].

The liver–heart axis is characterized by a bidirectional relationship wherein metabolic liver dysfunction may contribute to myocardial dysfunction, while cardiac dysfunction may, in turn, aggravate hepatic injury through hemodynamic mechanisms such as reduced perfusion and venous congestion. The inflammatory state associated with MASLD, marked by elevated CRP, chronic lipotoxicity, and aberrant hepatokine secretion, has been associated with structural and electrical cardiac remodeling and may contribute mechanistically to the development of HF and arrhythmias [75]. Atrial fibrillation (AF) represents a distinct cardiovascular phenotype associated with MASLD rather than simply an extension of MASLD-related ventricular remodeling. Observational studies have reported a higher incidence of cardiac arrhythmias, including AF, among individuals with MASLD [76]. Potential links include shared cardiometabolic risk factors, particularly obesity and type 2 diabetes, together with systemic inflammation, oxidative stress, atrial structural remodeling and fibrosis, and expansion or altered inflammatory activity of epicardial adipose tissue surrounding the atria [44,77]. These mechanisms provide biological plausibility for the observed association, although their relative contribution and independence from shared cardiometabolic risk factors remain uncertain.

Among the hepatokines implicated in liver–heart signaling, fibroblast growth factor 21 (FGF21) has received particular attention. It is a stress-induced hepatokine that enhances myocardial energy efficiency and mitigates fibrotic injury via the AMPK/PGC1-a pathway [78,79,80,81]. However, in chronic metabolic dysfunction, reduced β-Klotho expression can induce FGF21 resistance, neutralizing these cardioprotective mechanisms and perpetuating a cycle of systemic inflammation, plaque instability, and HF [80,82].

### 3.4. Hepatic Injury Caused by Cardiac Dysfunction

Cardiac dysfunction may adversely affect the liver through hemodynamic pathways, providing an important reverse component of the liver of MASLD–heart axis. Right-sided HF and elevated central venous pressure increase hepatic venous pressure and promote passive hepatic congestion, which may increase measured liver stiffness independently of underlying hepatic fibrosis [83]. Consequently, liver stiffness measurements should be interpreted cautiously in patients with HF, because congestion may influence elastography values; nevertheless, such measurements may provide complementary information regarding systemic congestion and prognosis [84,85].

In congestive hepatopathy, sustained elevation of hepatic venous pressure produces sinusoidal distension and congestion, which can impair hepatic microvascular perfusion and promote sinusoidal endothelial and hepatocellular injury [86]. Consequently, persistent congestion may lead to progressive vascular and extracellular-matrix remodeling, while sinusoidal injury, microthrombotic changes, and repetitive tissue damage may contribute to the development of congestive fibrosis and, in advanced cases, cirrhosis [87].

In addition to venous congestion, reduced cardiac output may compromise hepatic perfusion and oxygen delivery, particularly during acute or advanced HF. When sufficiently severe, this hemodynamic impairment may result in acute cardiogenic liver injury or hypoxic hepatitis, characterized by predominantly centrilobular hepatocellular injury [51]. Importantly, hepatic congestion and hypoperfusion frequently coexist rather than representing isolated mechanisms; pre-existing venous congestion may increase hepatic susceptibility to an acute reduction in blood flow and oxygen delivery [51]. Thus, both backward failure, through elevated venous pressures, and forward failure, through impaired systemic and hepatic perfusion, contribute to the reverse heart-to-liver component of the interaction.

## 4. Vascular Dysfunction in MASLD

Several observational and population-based studies have consistently associated MASLD with vascular dysfunction and increased cardiovascular risk even after adjustment for conventional cardiometabolic risk factors. However, the substantial overlap of MASLD with obesity, diabetes, hypertension, dyslipidemia, and other metabolic abnormalities should be considered when interpreting these findings, and causal independence cannot be inferred from statistical adjustment alone [88,89].

### 4.1. Endothelial Dysfunction and Vascular Injury

The vascular endothelium functions as a dynamic organ that regulates vascular tone, permeability, inflammation, and coagulation, thereby contributing to cardiovascular homeostasis. A key component of endothelial homeostasis is the production of nitric oxide (NO) by endothelial nitric oxide synthase (eNOS), which promotes vasodilation and inhibits platelet activation and aggregation [90]. Endothelial function is also critically influenced by flow shear stress (FSS), the frictional force generated by circulating blood on the vascular wall. Stable laminar FSS promotes a quiescent and vasoprotective endothelial phenotype, whereas disturbed or low-magnitude FSS, particularly at vascular bifurcations and in cardiometabolic conditions such as diabetes and hypertension, favors oxidative stress, inflammation, and prothrombotic signaling [91,92].

In liver disease, particularly MASLD and cirrhosis, these regulatory mechanisms may become disrupted, promoting endothelial dysfunction (ED) [93]. Chronic hepatic inflammation and lipid accumulation are associated with increased circulating pro-inflammatory mediators, including TNF-α and IL-6, together with enhanced reactive oxygen species (ROS) production [94]. These processes reduce NO bioavailability and promote endothelial activation, favoring a pro-inflammatory and prothrombotic phenotype [90]. Alterations in liver-derived mediators and adipokine signaling, including reduced adiponectin, may further contribute to oxidative stress and endothelial injury [95,96]. In cirrhosis, vascular dysfunction is particularly complex, as intrahepatic endothelial dysfunction and increased intrahepatic vascular resistance coexist with marked systemic and splanchnic vasodilation in the setting of portal hypertension [97]. Persistent endothelial dysfunction may contribute to vascular injury and remodeling, including increased arterial stiffness and the development of subclinical atherosclerosis [98].

Increased arterial stiffness, commonly assessed by pulse wave velocity, has been reported in chronic liver disease and MASLD and may reflect the combined effects of metabolic abnormalities, systemic inflammation, and vascular dysfunction within the liver–heart axis [99]. Endothelial dysfunction may therefore represent an important mechanistic interface linking liver disease with increased cardiovascular risk, including coronary artery disease and major adverse cardiovascular events [100].

Consistent with these mechanisms, endothelial dysfunction is considered an early feature of atherogenesis and has been frequently reported in individuals with MASLD [101]. Impaired endothelium-dependent vasodilation, increased vascular permeability, and a pro-inflammatory and prothrombotic endothelial phenotype have been described in this setting [93]. Clinical evidence supports these observations. In an Italian study assessing brachial artery vasodilation in response to ischemia, patients with MASLD exhibited impaired endothelium-dependent vasodilation compared with controls, and the association remained significant after adjustment for the reported confounders [102]. Furthermore, individuals with MASLD have been reported to exhibit reduced numbers and impaired function of circulating bone marrow-derived endothelial progenitor cells compared with matched controls [103], suggesting that diminished endothelial repair capacity may contribute to vascular disease progression. Higher circulating homocysteine concentrations have also been reported in MASLD and may further promote atherogenesis through oxidative stress and endothelial dysfunction [104,105].

Collectively, these findings support endothelial dysfunction as a potential bridge between hepatic metabolic and inflammatory abnormalities and vascular disease, linking mechanistic alterations in NO signaling and oxidative stress with clinically detectable abnormalities in vascular function and repair.

### 4.2. Arterial Stiffness

Arterial stiffness is a hallmark of early vascular aging and an independent predictor of cardiovascular events and all-cause mortality [106,107]. The significant association between MASLD and increased arterial stiffness suggests the involvement of common pathophysiological pathways linking hepatic and vascular dysfunction. MASLD has been associated with increased arterial stiffness across several clinical and population-based studies, consistent with the vascular consequences of the metabolic, inflammatory, and endothelial pathways discussed above [108,109,110].

In patients with MASLD, higher arterial stiffness and augmentation indices have been reported even in the early stages of MASLD and in patients without clinically apparent cardiovascular disease [3,111]. In young adults, a positive correlation between pulse wave velocity (PWV) and MASLD has been identified, suggesting shared pathophysiological mechanisms and highlighting arterial stiffness as a potential early marker of cardiometabolic risk [112].

### 4.3. Microvascular Dysfunction

Microvascular dysfunction is increasingly recognized as a key component of vascular pathology in MASLD, often subclinical, affecting coronary, peripheral, renal, and hepatic microcirculation. It may be associated with impaired tissue perfusion, reduced oxygen delivery, and increased vascular resistance [113,114]. The cardiovascular consequences of microvascular dysfunction may include angina, myocardial ischemia, and HF. Coronary microvascular dysfunction should be distinguished from epicardial coronary atherosclerosis. Whereas epicardial coronary atherosclerosis is characterized by plaque formation within the major coronary arteries, coronary microvascular dysfunction reflects functional and/or structural abnormalities of the small intramyocardial vessels and may impair coronary flow reserve and myocardial ischemia even in the absence of flow-limiting epicardial coronary stenosis [115]. Although the two processes may coexist and share metabolic, inflammatory, and endothelial risk pathways, they represent distinct vascular phenotypes and should not be considered interchangeable [115]. The coexistence of MASLD and type 2 diabetes mellitus (T2DM) has also been associated with a greater burden of microvascular and macrovascular complications, consistent with their shared cardiometabolic risk profile [116]. MASLD is also associated with retinal microvascular changes, including reduced vessel density and a higher prevalence of diabetic retinopathy [117].

## 5. MASLD and Cardiac Structural Remodeling

Several metabolic, neurohormonal, vascular, and hemodynamic abnormalities that frequently coexist with MASLD—including insulin resistance, glucose and lipid dysregulation, sympathetic and renin–angiotensin–aldosterone system (RAAS) activation, atherosclerotic burden, and altered hepatic hemodynamics—may contribute to adverse cardiac remodeling. Clinical studies have reported associations between MASLD, or various degree of liver dysfunction, with abnormalities in cardiac structure and function (Table 1); however, the extent to which these relationships reflect liver-specific effects rather than shared cardiometabolic risk remains uncertain.

### 5.1. Left Ventricular Remodeling and Functional Impairment

Observational studies have associated MASLD with an increased risk of HF, particularly HF with preserved ejection fraction (HFpEF) [142]. Importantly, HF should not be regarded as a single MASLD-associated cardiac phenotype. The clinical and pathophysiological overlap appears particularly prominent for HFpEF, in which obesity, type 2 diabetes, visceral adiposity, systemic inflammation, endothelial and microvascular dysfunction, and increased ventricular–arterial stiffness frequently coexist and may contribute to concentric remodeling, myocardial stiffness, and impaired diastolic function. By contrast, HFrEF represents a more heterogeneous syndrome in which ischemic myocardial injury, cardiomyocyte loss, ventricular dilation, and distinct stage-dependent metabolic adaptations may have a greater role. Thus, mechanistic and structural observations related to MASLD-associated HFpEF should not be directly extrapolated to HFrEF [44,142]. Sex and menopausal status may further modify the relationship between MASLD and cardiovascular remodeling. MASLD is generally more prevalent in men than in premenopausal women, whereas the prevalence and risk of more advanced liver disease increase in women after menopause, consistent with changes in sex-hormone exposure, adipose-tissue distribution, insulin sensitivity, and inflammatory activity [143]. In parallel, women, particularly at older ages and after menopause, are disproportionately affected by HFpEF and may exhibit greater ventricular–arterial stiffening, concentric remodeling, and microvascular dysfunction [144]. These observations suggest that the interaction between metabolic liver disease and HFpEF may differ according to sex and hormonal status. However, sex-stratified analyses of cardiac structure, function, and cardiovascular outcomes in MASLD remain limited, and many available observational studies do not provide sufficiently detailed sex-specific estimates [143]. Dedicated prospective studies are therefore needed to determine whether sex and menopausal status independently modify cardiovascular phenotypes associated with MASLD. A range of metabolic, inflammatory, and hemodynamic abnormalities accompanying MASLD may contribute to changes in cardiac structure and function [136]. Subclinical functional and structural cardiac abnormalities have been reported even in individuals without overt cardiovascular disease [119,122,127,145]. In the Dallas Heart Study, higher hepatic triglyceride content was linked to smaller LV volumes, lower LV mass, and greater LV concentricity, while the UK Biobank analysis similarly reported lower LV volumes and increased concentric remodeling in individuals with fatty liver disease [121,122]. The MESA study also demonstrated an association between liver fat burden and subclinical structural and functional cardiac abnormalities [128]. In the CARDIA study, NAFLD was associated with impaired left ventricular relaxation, higher filling pressures, worse left ventricular strain, and lower ejection fraction [118]. Other cohorts have reported greater LV wall thickness and mass in patients with MASLD, particularly among those with more severe steatosis or liver fibrosis [123]. Certain MASLD phenotypes have also been associated with thicker ventricular walls, impaired diastolic indices, and larger cardiac chambers [124]. Impaired LV GLS in MASH and MASH with fibrosis, has been reported, supporting the presence of early subclinical myocardial involvement [125]. In addition, a meta-analysis of 31 observational studies reported a significant association between MASLD and diastolic dysfunction, with higher E/e′ ratios, larger left atrial volume index, and greater odds of diastolic dysfunction [138]. Taken together, these studies suggest that MASLD is associated with heterogeneous patterns of subclinical cardiac remodeling and functional impairment, with reported phenotypes including increased concentricity, altered LV volumes, and variable changes in LV mass and wall thickness. These differences may reflect variation in study populations, metabolic burden, obesity, diabetes, and the severity of hepatic steatosis and fibrosis [120,122,126].

### 5.2. Myocardial Steatosis and Fibrosis

MASLD is associated with ectopic fat deposition in cardiac fat depots, including pericardial and epicardial adipose tissue [146]. Pericardial fat volume has been shown to be associated with arrhythmias, including atrial fibrillation, as well as with cardiovascular events [147]. Similarly, greater epicardial adipose tissue has been associated with subclinical atherosclerosis and cardiovascular events [148,149]. The association between MASLD and epicardial fat thickness has been documented with greater epicardial fat thickness correlating with more severe hepatic steatosis; in some studies, this association persisted after adjustment for age, waist circumference, body mass index, and conventional cardiovascular risk factors [138,150,151,152,153]. Greater epicardial adipose tissue has also been associated with coronary artery calcification [154]. Furthermore, experimental and translational evidence suggests that the local inflammatory and paracrine activity of epicardial adipose tissue may contribute to myocardial hypertrophy and fibrosis [155]. Intramyocardial lipid accumulation, referred to as myocardial steatosis, has been associated with abnormalities in myocardial contractile function and cardiac conduction, while mechanistic studies support a potential role of lipotoxicity in these processes [156,157].

The quantification of myocardial extracellular volume can serve as an indirect marker of myocardial fibrosis. In a cross-sectional study of patients with MASLD, liver fibrosis was associated with higher myocardial ECV, used as an indirect marker of myocardial fibrosis (r = 0.49, *p* < 0.001) [158]. Another study evaluated the relationship between liver fibrosis, assessed using the Enhanced Liver Fibrosis (ELF) test, and markers of myocardial injury, reporting associations between higher ELF values and higher concentrations of high-sensitivity cardiac troponins T and I. However, these associations were attenuated after adjustment for cardiovascular risk factors, suggesting that shared cardiometabolic determinants may partly account for the relationship between hepatic fibrosis and myocardial injury [159].

## 6. Clinical Implications

The growing overlap between MASLD and HF has important implications for risk stratification and prevention. In clinical practice, this supports earlier identification of hepatic disease in patients with cardiovascular risk factors and selective cardiovascular evaluation in individuals with MASLD according to symptoms, clinical findings, comorbidities, and overall cardiovascular-risk profile, rather than routine cardiac imaging or biomarker testing in all patients. For hepatic fibrosis risk stratification, a sequential non-invasive approach is recommended. The Fibrosis-4 (FIB-4) index, calculated from age, aspartate aminotransferase (AST), alanine aminotransferase (ALT), and platelet count, is primarily used to estimate the probability of advanced liver fibrosis rather than cardiovascular risk. In adults with an appropriate clinical risk profile, a FIB-4 value <1.3 generally identifies a low probability of advanced fibrosis, whereas values above the lower threshold warrant further assessment with vibration-controlled transient elastography (VCTE), the Enhanced Liver Fibrosis (ELF) test, or specialist evaluation as appropriate. FIB-4 should nevertheless be interpreted in the context of age and clinical status: its diagnostic accuracy is limited in adults younger than 35 years, a higher lower threshold of 2.0 is recommended in individuals older than 65 years, and the score should not be used for fibrosis risk stratification during acute illness [9,160]. Cardiovascular assessment should be individualized according to symptoms, physical findings, comorbidities, and overall cardiovascular-risk profile. Echocardiography and natriuretic peptide testing may be appropriate when HF or structural cardiac disease is clinically suspected or when the pre-test probability is sufficiently high, rather than being performed routinely in all individuals with MASLD [9,77]. For cardiovascular risk estimation, the American Heart Association PREVENT equations provide contemporary estimates of 10- and 30-year risk of total cardiovascular disease, including atherosclerotic cardiovascular disease and HF, in adults without established cardiovascular disease. The equations were developed and externally validated using large, diverse US cohorts and incorporate cardiovascular, renal, and metabolic variables rather than being derived from a bariatric or liver-disease-specific population. PREVENT may therefore complement clinical cardiovascular risk assessment in individuals with MASLD, although it is not a MASLD-specific risk score [161,162].

Lifestyle intervention remains the cornerstone of prevention. Achieving weight loss is one of the main therapeutic goals, since the hepatic response to weight reduction appears dose-dependent. Current guidelines recommend a sustained weight loss of ≥5% to reduce liver fat, 7–10% to improve hepatic inflammation, and ≥10% to increase the likelihood of fibrosis improvement. Greater weight loss is also associated with a higher probability of MASH resolution, although individual histological responses vary [9,163]. These liver-related benefits are accompanied by cardiovascular advantages, as evidence from large cohort studies associate the weight loss with lower the risk of HF [163,164,165]. Beyond weight reduction, dietary quality is an important component of lifestyle management. A Mediterranean-style dietary pattern, emphasizing vegetables, fruits, legumes, whole grains, nuts, olive oil, and fish, while limiting ultra-processed foods rich in added sugars and saturated fat and avoiding sugar-sweetened beverages, is recommended in individuals with MASLD. Such dietary patterns are associated with hepatic and cardiovascular benefits, although evidence that diet quality alone improves long-term liver-related clinical outcomes remains limited. Alcohol intake should also be routinely assessed, including its amount and pattern of consumption. Individuals with MASLD should be discouraged from alcohol consumption, particularly at moderate or high levels, while complete abstinence is recommended in patients with advanced fibrosis or cirrhosis [9]. Physical activity should complement dietary interventions, with current guidelines recommending preferably >150 min per week of moderate-intensity or 75 min per week of vigorous-intensity physical activity. Importantly, exercise can reduce hepatic steatosis and improve cardiometabolic fitness even in the absence of clinically significant weight loss. Both aerobic and resistance exercise have demonstrated hepatic and metabolic benefits, and combined aerobic and resistance training is generally preferred when a structured exercise program is prescribed; however, no single exercise modality has been established as superior for improving liver fibrosis or histological outcomes [9]. In addition, analyses across multiple cohorts have demonstrated that higher physical activity levels are associated with a statistically significant reduced risk of HF, particularly HFpEF when compared with HFrEF [166]. Of special interest are the results from a study designing an intensive lifestyle program combining both these interventions added to behavioral support proved a reduction in the HFpEF risk at the participants who achieved substantial weight reduction, an effect linked to reductions in visceral adiposity [167,168] further reinforcing the efficacy of structured and supervised exercise programs on a variety of cardiometabolic parameters [169,170]. Overall, sustained lifestyle modification represents a foundational strategy for slowing MASLD progression while also reducing future HF risk. However, maintaining these changes over the long term remains challenging, even under close monitoring and supervision, which highlights the need for additional medical therapeutic strategies.

## 7. Therapeutic Perspectives

Particularly lifestyle modification and therapies directed at established cardiometabolic comorbidities for treatment of MASLD, are supported by guideline recommendations and cardiovascular outcome data. Several newer pharmacological approaches have also demonstrated improvements in hepatic fat, biochemical markers, or histological endpoints but have not yet been shown to reduce cardiovascular events specifically in patients with MASLD.

### 7.1. Lifestyle and Weight-Loss Strategies

Weight reduction through dietary modification and increased physical activity improves hepatic steatosis and cardiometabolic risk factors, while greater and sustained weight loss may also improve steatohepatitis and fibrosis [9]. The therapeutic relevance extends beyond the liver because weight loss improves blood pressure, glycemic control, lipid profile, functional capacity, and overall cardiovascular risk. However, the evidence should not be interpreted as demonstrating that lifestyle-induced improvement of MASLD itself independently reduces cardiovascular events.

### 7.2. GLP-1 Receptor Agonists

For GLP-1 receptor agonists treatment there is favorable evidence for both cardiovascular outcomes and liver-related endpoints, although these data originate largely from different trial populations. In the SELECT trial, semaglutide 2.4 mg reduced major adverse cardiovascular events in 17,604 individuals with overweight or obesity and established cardiovascular disease but without diabetes [171]. Additionally, in the phase 3 ESSENCE trial, semaglutide improved both MASH resolution without worsening of fibrosis and fibrosis improvement without worsening of MASH in patients with biopsy-confirmed MASH and F2–F3 fibrosis [172]. However, there are not yet confident data whether improvement in MASH itself mediates cardiovascular risk reduction.

### 7.3. FGF-21-Based Therapies

FGF21 analogs represent a promising therapeutic strategy at earlier stage of the disease. Pegozafermin improved fibrosis and steatohepatitis-related histological endpoints in a phase 2b randomized trial involving patients with non-cirrhotic NASH and F2–F3 fibrosis [173]. FGF21 analogues influence lipid metabolism, insulin sensitivity, and energy homeostasis. Nevertheless, the current evidence remains primarily hepatic and metabolic and there are no confident data on the cardiovascular impact and outcome of this treatment.

### 7.4. Other Emerging Metabolic Therapies

Tirzepatide a dual GIP and GLP-1 receptor agonist produced substantial MASH resolution and fibrosis improvement in the phase 2 SYNERGY-NASH trial [173], but cardiovascular-outcome evidence specifically linking these hepatic improvements to fewer cardiovascular events in MASLD is not established.

Resmetirom, a liver-directed thyroid hormone receptor-β agonist, improved both steatohepatitis resolution and fibrosis in the phase 3 MAESTRO-NASH trial and also reduced LDL cholesterol [174].

The SGLT2 inhibitors cardiovascular and renal benefits are firmly established in diabetes, chronic kidney disease, and heart failure; for example, dapagliflozin reduced worsening heart failure or cardiovascular death in DAPA-HF [175]. Thus, SGLT2 inhibitors should be prescribed according to established cardiometabolic indications rather than considered specific MASLD-directed therapies on the basis of current evidence.

### 7.5. Integrated Cardiohepatic Treatment Model

An integrated cardiohepatic strategy should therefore be based on treatment of proven risk rather than on the assumption that therapies improving liver parameters reduce also cardiovascular events. Lifestyle modification and aggressive management of obesity, diabetes, hypertension, dyslipidemia, smoking, and established cardiovascular disease remain central [9]. GLP-1 receptor agonists and SGLT2 inhibitors may offer particular advantages when conventional cardiovascular or metabolic indications coexist because their cardiovascular benefits are supported by randomized outcome trials.

## 8. Future Directions and Research Gaps

### 8.1. Need for Prospective Studies

A key limitation across the current bibliography is the predominant reliance on observational, retrospective, or cross-sectional designs [176]. While these studies have been important in identifying significant associations, they lack the capacity to establish causality [99]. Longitudinal cohort studies are needed to determine the chronological progression from hepatic fat accumulation to subclinical vascular and myocardial damage.

Furthermore, regarding novel therapeutic interventions like glucagon-like peptide-1 receptor agonists (GLP-1RAs) and fibroblast growth factor 21 (FGF21) analogs, there is a clear demand for large-scale, multicenter, prospective randomized controlled trials (RCTs) [78,177]. Future prospective trials must have longer follow-up periods to evaluate the durability of treatment effects, assess potential long term adverse events, and monitor for disease relapse or rebound effects after treatment ending [78].

### 8.2. Mechanistic Gaps

Despite the established associations between liver and cardiovascular dysfunction, the precise molecular and pathophysiological pathways are not fully understood. For instance, while MASLD is consistently associated with chronic low-grade inflammation, insulin resistance, and oxidative stress additional research is required to fully map these pathophysiological networks [139]. Similarly, the mechanisms linking obesity to impaired right ventriculo-pulmonary arterial (RV-PA) coupling and right ventricular remodeling remain incompletely understood [178].

From a pharmacological perspective, the exact vasculoprotective mechanisms of newer agents, such as semaglutide, extend beyond mere metabolic improvement. However, the presence and role of GLP-1 receptors on endothelial cells remain a subject of ongoing debate, representing a key mechanistic gap that future basic and translational research needs to bridge. Additionally, the cellular and vascular foundations of the “obesity paradox” in ischemic cardiomyopathy—where obesity paradoxically associates with improved HF prognosis despite promoting arterial stiffness— remain unresolved [179].

### 8.3. Standardization of Screening and Endpoints

A challenge in synthesizing data across current studies is the heterogeneity in diagnostic criteria and methodology. In evaluating MASLD, diagnostic tools vary widely from non-invasive clinical scores (e.g., Fatty Liver Index) and ultrasonography to advanced computed tomography (CT), magnetic resonance imaging (MRI), and liver biopsy [99,139]. Standardizing diagnostic screening algorithms is essential for future research. This is particularly crucial given that standard imaging faces severe technical limitations in patients with severe adiposity, necessitating the refinement or development of novel imaging techniques tailored for obese populations [178].

Furthermore, clinical trials frequently rely on surrogate endpoints, such as changes in hepatic fat fraction via imaging or specific echocardiographic parameters of diastolic function. Future research must standardize and prioritize “hard” clinical outcomes, ensuring a clear, validated correlation between these markers and long-term, clinical endpoints such as liver-related morbidity, cardiovascular events, and overall mortality [78]. Finally, the standardization of adiposity markers is required, relying solely on BMI introduces accuracy limitations in HF cohorts, highlighting the need to integrate alternative markers of fat distribution, such as visceral adiposity or waist-to-hip ratio, into future trial designs [179].

### 8.4. Precision Medicine Opportunities

The heterogeneous response of patients to metabolic therapies underscores the growing importance of precision medicine. Meta-analytic data have revealed differences in drug efficacy across patient subgroups; for example, FGF21 analogs demonstrate significant efficacy in non-cirrhotic populations but have not shown significant improvement in patients with established cirrhosis [78]. This distinct response highlights the need to discover and validate specific biomarkers that can accurately predict therapeutic responsiveness based on the exact stage of liver disease [78].

Future strategies should move away from a “one-size-fits-all” approach, examine individual metabolic profiles, disease severity, inflammatory status, and genetic background to design personalized interventions. In the context of advanced HF and ischemic cardiomyopathy, precisely phenotyping patients according to their specific HF subtype (HFrEF, HFmrEF, or HFpEF) alongside their cardiometabolic risk profile will be essential to optimizing treatment efficacy, maximizing cardiovascular protection, and minimizing adverse events [179].

## 9. Conclusions

The MASLD–heart axis represents a complex bidirectional interaction involving metabolic dysfunction, chronic low-grade inflammation, oxidative stress, endothelial dysfunction, and hemodynamic alterations. Clinical evidence consistently associates MASLD with vascular dysfunction, adverse cardiac remodeling, HF, and atherosclerotic cardiovascular disease, while experimental and translational studies provide plausible biological mechanisms linking hepatic and cardiovascular abnormalities. Although several clinical associations persist after adjustment for conventional cardiometabolic risk factors, shared determinants and residual confounding limit definitive causal attribution. Recognizing this interplay supports integrated cardiovascular and hepatic risk assessment, early screening, and multidisciplinary management. Lifestyle modification remains the cornerstone of therapy, while emerging metabolic treatments offer promising opportunities to improve both hepatic and cardiovascular outcomes. Future prospective mechanistic and interventional studies are needed to establish causal pathways, validate biomarkers, and develop precision medicine strategies.

## Figures and Tables

**Table 1:** Studies evaluating the association of MASLD/NAFLD with cardiac structure and function.

Authors (Year of Publication)	Study Type, Population (*N*)	Liver Assessment Method	Principal Adjustment	Main Cardiac Structure and Function Findings
VanWagner et al. (2015) [118]	CARDIA cohort, community-based adults (*N* = 2713)	CT	Demographic and cardiovascular risk factors; BMI examined separately	Higher LV mass/wall thickness and adverse systolic/diastolic indices.
Chiu et al. (2020) [119]	Framingham Heart Study; cross-sectional, community adults (*N* = 2356)	CT	Age, sex, cohort, cardiovascular risk factors; additional BMI adjustment	Higher LV mass and E/e′; associations attenuated after BMI adjustment.
VanWagner et al. (2020) [120]	CARDIA cohort; longitudinal analysis (*N* = 1827)	CT	HF risk factors and change in BMI; BMI sensitivity adjustment	Adverse LV remodeling, impaired relaxation and worse GLS; BMI attenuated associations.
Jamialahmadi et al. (2023) [121]	UK Biobank cohort, middle-aged and older adults (*N* = 18,848)	MRI-PDFF	Demographic and cardiometabolic factors	Altered LV structure, including lower LV volumes and LV mass.
Saha et al. (2024) [122]	Dallas Heart Study cohort, community-based adults	Spectroscopy; hepatic triglyceride content quantification	Multivariable cardiometabolic adjustment	Higher liver fat associated with adverse LV geometry/remodeling.
Peng et al. (2022) [123]	Adults with MASLD/MAFLD (*N* = 228)	Steatosis plus cardiometabolic risk factors; transient elastography/CAP	Metabolic risk factors	Impaired LV diastolic function and adverse cardiac morphology.
Wang et al. (2025) [124]	Chinese adults; cohort	MASLD phenotypes by data-driven clustering	Multivariable phenotype-based analysis	Higher-risk MASLD clusters associated with adverse cardiac remodeling.
Hüning et al. (2025) [125]	Adults with liver disease; observational	Noninvasive hepatic inflammation/fibrosis assessment	Multivariable clinical adjustment	Greater inflammation/fibrosis associated with worse GLS.
Lin et al. (2025) [126]	Patients with type 2 diabetes; observational	MASLD plus noninvasive fibrosis stratification	Multivariable cardiometabolic adjustment	Fibrosis, but not MASLD alone, associated with LV concentric hypertrophy.
Simon et al. (2022) [127]	Swedish cohort with biopsy-proven NAFLD (*N* = 10,422)	Liver biopsy	Matched/multivariable cardiovascular risk adjustment	Higher cardiovascular event rates with NAFLD.
Kusner et al. (2024) [128]	MESA; community-based cohort	Imaging-based liver fat relative to body/visceral fat	Cardiometabolic risk adjustment	Disproportionate liver fat associated with HF markers and LV abnormalities.
Cassidy et al. (2015) [129]	Cross-sectional; type 2 diabetes/NAFLD/control groups (*N* = 57)	Imaging-based steatosis/1H-MRS	Group comparison/matching	More frequent LV structural and functional abnormalities with NAFLD.
Lee et al. (2018) [130]	Cross-sectional, patients with NASH	Histology	Clinical/metabolic adjustment	NASH associated with subclinical myocardial dysfunction (GLS/strain).
Fallo et al. (2009) [131]	Cross-sectional, essential hypertension (*N* = 86)	Ultrasound	Metabolic variables/insulin resistance	NAFLD associated with LV diastolic dysfunction.
Jung et al. (2017) [132]	Cross-sectional, Korean general population (*N* = 20,821)	Ultrasound	Demographic and cardiometabolic covariates	Impaired LV diastolic function and altered LV geometry.
Aksu et al. (2021) [133]	Clinical cross-sectional, non-obese NAFLD patients (*N* = 74)	Ultrasound	Limited multivariable adjustment	Early LV diastolic and structural abnormalities.
Mantovani et al. (2015) [134]	Cross-sectional, patients with type 2 diabetes (*N* = 222)	Ultrasound	Hypertension and indexed LV mass; BMI/age/sex interactions examined	NAFLD independently associated with early LV diastolic dysfunction.
Sonaglioni et al. (2022) [135]	Observational, non-advanced NAFLD without overt heart disease (*N* = 92)	Transient elastography	Multivariable logistic regression	Higher liver stiffness associated with impaired LV-GLS.
Chang et al. (2025) [136]	Type 2 diabetes and NAFLD; cross-sectional (*N* = 97)	NAFLD severity by ultrasound	Adjusted analyses for confounders	Moderate-severe NAFLD associated with impaired left atrial function.
Lai et al. (2022) [137]	Observational, Asian population (*N* = 2161)	NAFLD and fibrosis assessment	Traditional risk factors and epicardial fat	Fibrosis associated with adverse atrial mechanics and diastolic indices.
Yong et al. (2022) [138]	Meta-analysis of observational studies	NAFLD across included studies	Study-level adjusted estimates where available	NAFLD associated with adverse cardiac structural and functional changes.
Goliopoulou et al. (2023) [139]	Systematic review and meta-analysis	NAFLD across included studies	Study-level adjusted estimates where available	NAFLD associated with impaired LV diastolic function.
Gohil et al. (2023) [140]	Systematic review	NAFLD across included studies	Not applicable	Evidence supports an association with LV diastolic dysfunction.
Gruber et al. (2025) [141]	Systematic review and meta-analysis	NAFLD across included studies	Study-level adjusted estimates where available	NAFLD associated with impaired GLS and myocardial strain.

NAFLD, NASH, and FLD terminology was retained where used in the original studies. MASLD, metabolic dysfunction-associated steatotic liver disease; NAFLD, non-alcoholic fatty liver disease; NASH, non-alcoholic steatohepatitis; FLD, fatty liver disease; LV, left ventricular; T2D, type 2 diabetes; CT, computed tomography; MRI-PDFF, magnetic resonance imaging proton density fat fraction; GLS, global longitudinal strain.

## Data Availability

No new data were created or analyzed in this study. Data sharing is not applicable to this article.
